# Rational Construction of Bi_2_CuO_12_Se_4_ and VGCFs@Fe_2_O_3_ Composite Electrodes for High‐Performance Semi‐Solid‐State Asymmetric Supercapacitors

**DOI:** 10.1002/smtd.202400149

**Published:** 2024-06-16

**Authors:** Manchi Nagaraju, Bhimanaboina Ramulu, Shaik Junied Arbaz, Edugulla Girija Shankar, Ampasala Surya Kiran, Jae Su Yu

**Affiliations:** ^1^ Department of Electronics and Information Convergence Engineering Institute for Wearable Convergence Electronics Kyung Hee University 1732 Deogyeong‐daero, Giheung‐gu Yongin‐si Gyeonggi‐do 17104 Republic of Korea

**Keywords:** bimetallic selenide, cycling stability, electrodeposition, ultra‐thin nanosheets, vapor‐grown carbon fibers

## Abstract

Recently, supercapacitors (SCs) are extensively explored as effective energy storage devices. Specifically, asymmetric SCs are being developed to enhance energy density using suitable materials with favorable nanostructures. This study describes the construction of a bismuth copper selenite (BCS‐200) working electrode with an ultrathin nanosheet (UTNS) architecture. This morphology is achieved using a low‐cost electrodeposition (ED) method, followed by annealing. The impact of ED time on the development of morphology is studied by synthesizing comparative electrodes simultaneously. The optimized BCS‐200 electrode prepared with a deposition time of 200 s shows higher specific capacity/capacitance (C_s_/C_sc_) values of 330.9 mAh g^−1^/2206.6 F g^−1^ than the other synthesized electrodes (BCS‐100, BCS‐150, BCS‐250, and BCS‐300). Besides, a vapor‐grown carbon fiber (VGCF)‐added Fe_2_O_3_ composite coated on nickel foam (NF) is developed as a negative electrode. The VGCFs@Fe_2_O_3_/NF electrode exhibits the (C_s_/C_sc_) values of 183.5 mAh g^−1^/734.4 F g^−1^, which is associated with ultra‐high cycling stability. In addition, the fabricated BCS‐200 and VGCFs@Fe_2_O_3_/NF electrodes are combined to construct a wearable semi‐solid‐state asymmetric SC (SSASC) with an energy density (E_d_) of 20.5 Wh kg^−1^ and a cycling stability of 91.7% over 40000 charge/discharge cycles. Furthermore, the real‐time applicability of the SSASC is verified by powering it in practical applications.

## Graphical abstract

The BCS‐200/VGCFs@Fe_2_O_3_/NF semi‐solid‐state asymmetric supercapacitor demonstrated an energy density of 20.5 Wh kg^−1^ and 91.7% cycling stability over 40000 cycles and successfully powered various portable and wearable electronics using green energy.

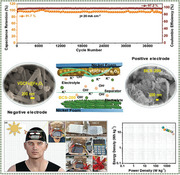

## 1 Introduction

Wearable electronic devices are gaining much attention in the fields of biomedicine, health monitoring, and electronic sensors owing to their portability, compactness, and compatibility. Solid‐state and wearable supercapacitors (SCs) are often combined with gel electrolytes, to prevent leakage and packing issues.^[^ ^1^, ^2^, ^3^, ^4^ ^]^ In the field of energy storage devices, SCs have demonstrated many advantages over lithium (Li)‐ion batteries and fuel cells, including rapid charging, low risk‐factors, high power density (P_d_), low maintenance, and extended cycling life.^[^ ^5^, ^6^ ^]^ However, SCs have notable disadvantages such as low energy density (E_d_), unwieldy redox behavior, and low voltage window compared to batteries.^[^ ^7^, ^8^, ^9^ ^]^ Based on the energy density equation (E_d_ = 1/2 × CV^2^), the issue of low energy density can be resolved by increasing the capacitance (C) and operating voltage (V).^[^ ^10^, ^11^, ^12^ ^]^ Therefore, there are two ways in which the E_d_ of SC devices can be increased. The first method involves creating large operating voltage windows that can be obtained by constructing hybrid or asymmetric SCs.^[^ ^13^, ^14^ ^]^ The second approach is to design novel electrode materials with key features, such as a large surface area, highly redox‐active compounds, low internal resistance, and nanostructures that favor electrochemical kinetics, all of which can improve the specific capacitance (C_sc_) of SCs. The optimal solution is to adopt both approaches simultaneously.

Thus far, for the preparation of SCs, different types of electrode materials such as metal hydroxides, single/binary transition metal oxides, sulfides/selenides, and phosphates have been reported in several previous studies.^[^ ^15^, ^16^ ^]^ Among them, binary transition metal selenides (TMSs), such as NiFe_2_Se_4_, Bi‐Co‐Se, NiCo_2_Se_4_, Zn‐Co‐Se, Co‐Se‐Mo, and (Ni, Co)_0.85_Se, have gained much attention in the field of SCs because of their numerous active sites, tunable morphology, rate capability, and excellent theoretical capacity/capacitance compared to single TMSs.^[^ ^17^ ^]^ Expressly, the bismuth (Bi)‐based electrode materials have been used in various fields such as SCs, rechargeable batteries, catalysis, and biosensors applications, owing to the advantages of quasi‐conversion reaction mechanism (Bi_2_O_3_ ⇔ Bi^0^) in an aqueous alkaline medium, low toxicity, and cost‐effectiveness.^[^ ^18^, ^19^, ^20^ ^]^ Furthermore, the Bi‐based electrode materials reveal large theoretical capacitance value, negative potential window, stable redox reactions, and decent electrochemical stability.^[^ ^21^ ^]^ On the other hand, copper (Cu)‐based materials have narrow energy band gap, high earth abundance, reasonable price, ease of synthesis, and facile morphological tunability. Metal copper (Cu), Cu_2_O, and CuO are promising candidates for electrochemical energy storage devices such as batteries and SCs. The Cu‐based electrode materials showed high theoretical capacitance (1800 F g^−1^) and notable electrochemical response in aqueous electrolytes.^[^ ^22^ ^]^ However, they are suffering from poor electrical conductivity, which led to slow kinetics, poor rate capability, and low surface area.^[^ ^23^, ^24^ ^]^

Meanwhile, selenium (Se), oxygen (O), and sulfur (S) share many properties depending on their positions in the periodic table. Among them, the Se exhibits a higher electrical conductivity (1 × 10^−3^ S m^−1^) and a lower energy gap than O and S.^[^ ^25^, ^26^, ^27^ ^]^ So far, various research groups have reported TMS‐based electrodes for SC applications. V. T. Chebrolu et al. prepared a NiCu(OH)_2_@Ni‐Cu‐Se electrode, which revealed a C_sc_ value of 264.91 F g^−1^ and a cycling retention of 75.8% after 3000 GCD cycles.^[^ ^6^ ^]^ J. Xia et al. prepared a NiCo_2_Se_4_ electrode, exhibiting a high C_sc_ value of 1874.6 F g^−1^ and a cycling retention of 89.4% after 5000 GCD cycles.^[^ ^28^ ^]^ P. M. Anjana et al. reported a NiCo_2_Se_4_ electrode and it revealed a high C_sc_ value of 820 F g^−1^ and a good cycling retention of 95% after 10000 GCD cycles.^[^ ^29^ ^]^ W. Wu et al. demonstrated a CuCoSe@NiS electrode with a high C_sc_ value of 2937.6 F g^−1^ and a decent cycling retention of 98.8% after 1000 GCD cycles.^[^ ^30^ ^]^

Several synthetic techniques such as hydrothermal/solvothermal methods, microwave methods, sol‐gel synthesis, green synthesis, selenization, and electrodeposition (ED) have been adopted for the synthesis of TMSs as active materials. Of these methods, the chronoamperometry‐based ED process is an easy, rapid, and cost‐effective technique for the preparation of working electrodes for SCs.^[^ ^31^, ^32^ ^]^ In addition to the fabrication technique, the morphology of the working material/electrode is prominent in terms of electrochemical performance. Using different synthesis methods, researchers have fabricated several types of nanostructures, such as nanoparticles, nanowires, nanosheets (NSs), nanorods, and nanobelts.^[^ ^33^ ^]^ Among them, two‐dimensional (2D) NSs have attracted much attention because of their unique advantages of large surface area, high electrode‐electrolyte interaction, increased charge accommodation, low resistance diffusion paths, high voltage window, and high electrical conductivity. Considering these structural and above benefits, we synthesized ultrathin NSs (UTNSs) constructed from BiCuSe (BCS), which can serve as a positive electrode. The resultant electrode material revealed higher energy density value, extended the potential window, and enhanced energy storage properties.

Recently, different negative electrode materials have been used to fabricate asymmetric hybrid SCs. Carbonaceous materials such as activated carbon, carbon nanotubes, graphite, graphene, vapor‐grown carbon fibers (VGCFs), bio‐extracted carbon, and metal oxides such as Fe_2_O_3_, Cu_2_O, Bi_2_O_3_, V_2_O_5_, and MoO_3_ are also considered negative electrode materials.^[^ ^34^, ^35^ ^]^ Especially, iron oxides are considered promising electrode materials because of their natural abundance, several active sites, low cost, high redox activity, high potential window, and environmental friendliness.^[^ ^36^, ^37^ ^]^ In particular, Fe_2_O_3_ shows good reversible oxidation/reduction owing to the interchangeability between Fe^3+^ and Fe^2+^ ions. Nevertheless, Fe_2_O_3_ suffers from the limited surface area, short life cycle, lack of ionic diffusion, and low electrical conductivity.^[^ ^38^, ^39^ ^]^ To overcome these disadvantages, the particle size can be reduced to significantly increase the surface area and boost electrochemical performance. Another approach is to combine carbonaceous materials to enhance the stability, conductivity, and overall performance.^[^ ^40^, ^41^, ^42^ ^]^ Among the aforementioned materials, VGCFs exhibit high thermal and chemical stabilities and mechanical rigidity. A composite negative electrode material can be obtained by combining redox‐based Fe_2_O_3_ with carbon‐based VGCFs to inherit the structural advantages of both materials.

In this study, we focused on improving the cycling stability and E_d_ of the fabricated SCs. Initially, a BCS‐200 (BCS electrodeposited for 200 s) electrode was synthesized on a nickel foam (NF) substrate using a facile ED process, followed by annealing for use as the positive electrode. Besides, VGCFs were also introduced into the pre‐synthesized Fe_2_O_3_ material to obtain a composite VGCFs@Fe_2_O_3_ negative electrode material with multiple merits. Both the positive and negative electrodes demonstrated good electrochemical properties and high cycling stabilities. A semi‐solid‐state asymmetric (SC) (SSASC) cell consisting of the BCS‐200 positive electrode, VGCFs@Fe_2_O_3_/NF composite negative electrode, and gel electrolyte was fabricated, which demonstrated enhanced energy density and elevated cycling stability. Furthermore, it has been used to power wearable electronic devices to verify their real‐time operation.

## 2 Results and Discussion

**Figure 1:**
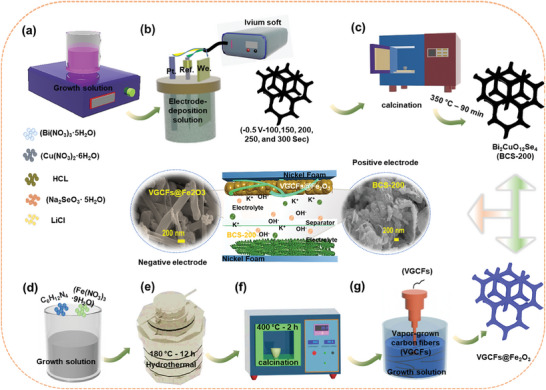
Schematic illustrations for the a–c) positive BCS‐200 and d–g) negative VGCFs@Fe_2_O_3_ composite electrode materials.

The preparation process for the BCS‐200 UTNSs and VGCFs@Fe_2_O_3_ composite electrode materials is schematically illustrated in **Figure** **1**. First, the 40 mL growth solution was prepared by dissolving the Bi(NO_3_)_3_·5H_2_O, Cu(NO_3_)_2_·6H_2_O, Na_2_SeO_3_·5H_2_O, and LiCl salts in a beaker. Later, HCl was added until the pH reached ≈3.5, as shown in Figure 1a. Next, the ED process was performed at an applied voltage (−0.5 V) with different deposition times of 100, 150, 200, 250, and 300 s, as shown in Figure 1b. During the ED process, The LiCl and HCl act as structure‐directing salt and reducing agents, respectively. During the ED process, the Na_2_SeO_3_·5H_2_O precursor in aqueous solution underwent hydrolysis, turned into selenous acid (H_2_SeO_3_), and subsequently reduced to Se^2−^ via the formation of Se by the applied electric field. Meanwhile, the Bi^0^/Bi^+3^ and Cu^0^/Cu^2+^ ions undergo uniform nucleation and are finally deposited as the BCS. The corresponding reactions are as follows:^[^ ^43^, ^44^ ^]^

(1) $$Na_{2}\mathrm{Se}O_{3}+xH_{2}O\to \;2\mathrm{Na}+\mathrm{Se}O_{2}+xH_{2}O$$

(2) $$\mathrm{Se}O_{2}+H_{2}O\to H_{2}\mathrm{Se}O_{3}$$

(3) $$H_{2}\mathrm{Se}O_{3}\;+\;4H^{+}+4e^{-}\to \mathrm{Se}\left(s\right)\;+3H_{2}O$$

(4) $$\mathrm{Se}\left(s\right)\;+\;2e^{-}\to Se^{2-}$$

(5) $$\mathrm{Bi}\;\mathrm{ions}\;+\;\mathrm{Cu}\;\mathrm{ions}\;+\;Se^{2-}\to \mathrm{BCS}$$

The BCS‐200 material forms UTNSs‐like nanostructures on the NF substrate. Later, the BCS‐200 material electrode was calcined in a furnace to remove hydroxides and impurities (Figure 1c). The Fe(NO_3_)_3_·9H_2_O and C_6_H_12_N_4_ were dissolved in 50 mL of mixed growth solution containing C_3_H_8_O_3_ and CH_3_CHOHCH_3_, as shown in Figure 1d. The solvothermal process was carried out at 180 °C for 12 h, as shown in Figure 1e. During the solvothermal process, hexamethylenetetramine (HMTA) was mixed with the Fe^+2^/Fe^+3^ ions which led to the formation of the Fe_2_O_3_ precursor. As well known, the HMTA acts as a surfactant which helps the aggregation of nanoparticles and promotes the formation of well‐dispersed nanospheres with a high surface area.^[^ ^44^ ^]^ The corresponding reactions as follows:^[^ ^45^ ^]^

(6) $$2\mathrm{Fe}{\left(NO_{3}\right)}_{3}\cdot 9H_{2}O\to Fe_{2}O_{3}+18H_{2}O+6NO_{2}+O_{2}$$

Later, the obtained Fe_2_O_3_ precursor was calcined in a furnace as shown in Figure 1f. During this process, the precursor was converted into the corresponding oxide in the presence of oxygen from the ambient air. Later, using the sonication process, the VGCFs were added to the Fe_2_O_3_ NSs as shown in Figure 1g. After that, the Fe_2_O_3_ NSs were embedded in uniformly dispersed VGCFs. Finally, a VGCFs@Fe_2_O_3_ composite electrode material was obtained.

**Figure 2:**
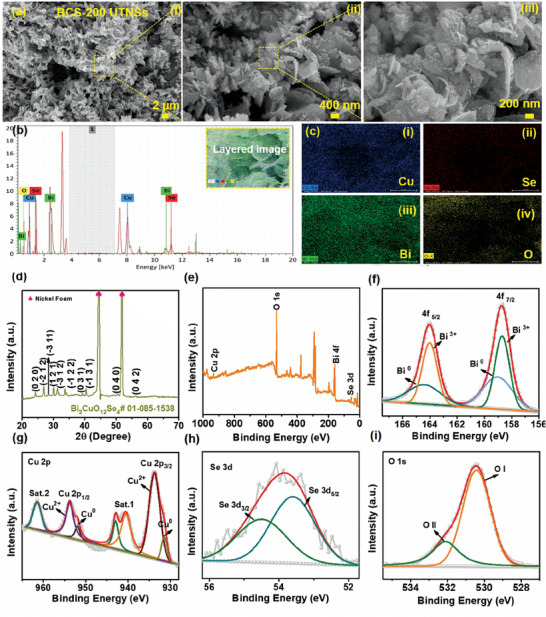
ai–iii) Low‐ and high‐magnification FE‐SEM images, b) EDX spectrum ci–iii) elemental mapping images of the BCS‐200 material. d) XRD pattern of the BCS‐200. e) Total XPS survey scan spectrum and HR XPS core‐level spectra of the f) Bi 4f, g) Cu 2p, h) Se 3d, and i) O 1s elements.

The surface morphology of the synthesized BCS‐200 sample was analyzed using field‐emission scanning electron microscopy (FE‐SEM), as shown in **Figure** **2**. The low‐magnification FE‐SEM image revealed that a large number of UTNSs were vertically arranged on the NF (Figure 2a(i)). The NF was fully covered by a large number of uniform UTNSs (Figure 2a(ii)). In addition, a high‐magnification FE‐SEM image displayed the vertical growth of UTNSs with a highly porous morphology (Figure 2a(iii)). The benefits of the 2D morphology are as follows: The 2D UTNSs structure may provide superior surface redox active sites, enabling rapid ion/electron transfer, which improves the electrochemical performance. Furthermore, the morphology during the charge/discharge process reduces the volume expansion, thereby enhancing the mechanical stability and cycling life. The elemental composition of BCS‐200 was characterized using energy‐dispersive X‐ray (EDX) spectroscopy, as shown in Figure 2b. The EDX spectrum revealed the presence of Bi, Cu, Se, and O, whereas the layered electronic image of the inset of Figure 2b showed an even distribution of these elements in the achieved morphology (Figure 2c(i)–(iv)). The FE‐SEM images of the BCS‐100, BCS‐150, BCS‐250, and BCS‐300 electrodes are shown in Figures S1 and S2 (Supporting Information). The phase and crystallinity of the synthesized BCS‐200 were investigated using X‐ray diffraction (XRD), as shown in Figure 2d. The peaks are at the 2θ values of 24.1°, 26.9°, 28.3°, 30.1°, 31.1°, 33.7°, 39.1°, 40.4°, 50.4°, and 56.3°, corresponding to the (0 2 0), (−2 1 2), (−3 1 1), (1 2 1), (−3 2 1), (−1 2 2), (0 3 1), (−1 3 1), (0 4 0), and (0 4 2) crystal planes of the Bi_2_CuO_10_Se_4_ phase (JCPDS # 01‐085‐1538), respectively. Moreover, two intense peaks can be seen at the 2θ values of 44.3° and 51.7° that are attributed to the NF substrate. No other crystal planes were observed, indicating the high purity of the prepared material. These significant peaks represented the stable bonding of Bi, Cu, and Se, which may synergistically influence the electrochemical characteristics.

In addition, the surface chemistry of the prepared BCS‐200 material and its valence states were studied by X‐ray photoelectron spectroscopy (XPS) analysis. The total XPS survey spectrum confirmed the presence of Bi 4f, Cu 2p, Se 3d, and O 1s in the prepared BCS‐200 material, as shown in Figure 2e. The atomic percentages of the BCS‐200 material were obtained from the XPS analysis, which is well consistent with the EDX results, as displayed in Table S1 (Supporting Information). The Bi 4f core‐level spectrum is shown in Figure 2f. Here, the Bi 4f spectrum consists of two peaks at the binding energies of ≈164.0 and 158.7 eV, which can be indexed to the Bi 4f_5/2_ and Bi 4f_7/2_ states, respectively. The Bi region is separated by an energy gap of 5.3 eV, indicating the presence of Bi^0^ in the Bi^3+^ oxidation state. Moreover, the Bi 4f_7/2_ peak is again divided into two sub‐peaks positioned at ≈159.1 and 158.6 eV which are assigned to the Bi (0) of the metallic state. Similarly, the Bi 4f_5/2_ peak is divided into two sub‐peaks positioned at ≈164.4 and 163.9 eV which are assigned as the Bi (III) state.^[^ ^19^, ^46^ ^]^ The core‐level Cu 2p spectrum is shown in Figure 2g. The Cu 2p spectrum exhibited two peaks at ≈933.8 and 958.7 eV, corresponding to Cu 2p_3/2_ and Cu 2p_1/2_, respectively. The difference was 25 eV, indicating the presence of Cu(II) ions in BCS‐200. The Cu 2p_1/2_ signal can be fitted into two peaks at 954.4 and 952.2 eV, which is ascribed to the Cu^2+^ and Cu^0^, respectively. The Cu 2p_3/2_ signal can also be fitted into two peaks at 934.5 and 932.3 eV, attributing to the Cu^2+^ and Cu^0^, respectively. Moreover, three satellite peaks were observed at 940.6, 942.9, and 961.4 eV. This indicates that Cu exists in the Cu(0)/Cu(II) oxidation states.^[^ ^47^, ^48^ ^]^ The core‐level Se 3d XPS spectrum is shown in Figure 2h. One peak was observed at ≈53.6 eV associated with Se 3d_5/2_ and another peak at ≈54.5 eV associated with Se 3d_3/2_ with a peak difference of 0.9 eV, thereby implying the presence of the Se^2−^ state.^[^ ^49^, ^50^ ^]^ The high‐resoluion (HR)‐XPS O 1s spectrum is shown in Figure 2i. The O 1s can be divided into two sub‐peaks at 530.4 and 532.1 eV which are attributed to metal‑oxygen bonding and adsorbed oxygen species, respectively.^[^ ^51^ ^]^ All of the above physical and chemical characterizations confirm that the prepared electrode material is in the Bi_2_CuO_10_Se_4_ phase.

**Figure 3:**
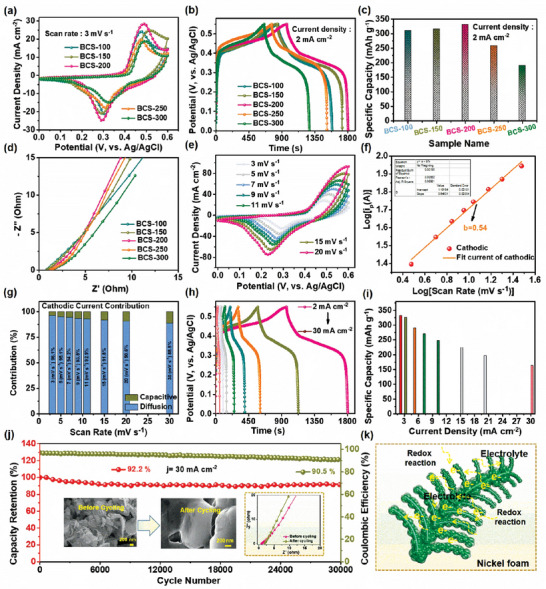
Comparative a) CV curves, b) GCD curves, c) C_s_ values, and d) EIS plots of the BCS‐100, BCS‐150, BCS‐200, BCS‐250, and BCS‐300 electrodes. e) CV curves at 3–20 mA cm^−2^ and f) b value, g) current contribution percentage at different scan rates, h) GCD curves, and i) C_s_ values of the BCS‐200 electrode. j) Cycling test and Coulombic efficiency results. The insets show the FE‐SEM images and EIS plots of the BCS‐200 electrode before and after the cycling test. k) Structural merits for the BCS‐200 UTNSs electrode material.

The electrochemical response of all the prepared BCS‐100, BCS‐150, BCS‐200, BCS‐250, and BCS‐300 electrodes was evaluated by cyclic voltammetry (CV), galvanostatic charge‐discharge (GCD), and electrochemical impedance spectroscopy (EIS) tests using the IviumStat electrochemical workstation (IviumStat Technologies). The three‐electrode system consists of active material loaded NF substrate as the working electrode, Pt wire as the counter electrode, Ag/AgCl as the reference electrode, and 1 M KOH (potassium hydroxide) aqueous electrolyte solution as the electrolyte. The CV profiles of the five electrodes were compared at a fixed 3 mV s^−1^ within the potential window of 0.0–0.6 V as shown in **Figure** **3**a. All the CV curves revealed a pair of redox peaks, signifying Faradaic behavior. The BCS‐200 electrode exhibited a higher CV response than the other electrodes. The GCD curves were compared at a fixed current density value of 2 mA cm^−2^ within the voltage window of 0.0‐0.55 V. The BCS‐200 electrode exhibited longer charge/discharge times than the BCS‐100, BCS‐150, BCS‐250 and BCS‐300 electrodes as shown in Figure 3b. The resultant high electrochemical performance is due to the advantages of the active materials that are directly grown on the NF. The NF increases the charge‐transfer rate and decreases the diffusion length between the electrolyte and electrode ions. Moreover, in the ED process, the prepared electrode does not require additives such as a binder, thus reducing the internal resistance and increasing the electrical conductivity. In addition, UTNSs offer several electroactive sites and rapid electron transmission between the electrode and electrolyte, which improves the electrochemical performance. Based on the GCD curves, the specific capacitance/capacity (C_sc_/C_s_) was calculated using Equations (S1) and (S2) (Supporting Information). The resultant C_s_ (C_sc_) values of the BCS‐100, BCS‐150, BCS‐200, BCS‐250, and BCS‐300 electrodes were found to be 309.5 mAh g^−1^ (2064.6 F g^−1^), 315.9 mAh g^−1^ (2110.2 F g^−1^), 330.9 mAh g^−1^ (2206.6 F g^−1^), 258.1 mAh g^−1^ (1717.3 F g^−1^), and 190.4 mAh g^−1^ (1272 F g^−1^), respectively, as shown in Figure 3c. In addition, for the five electrodes, the solution resistance (R_s_) and charge‐transfer resistance (R_ct_) were studied using EIS in the frequency range 0.01 Hz‐100 K Hz. The EIS plots of the BCS‐100, BCS‐150, BCS‐200, BCS‐250, and BCS‐300 electrodes showed the R_s_ values of 1.17, 0.92, 0.87, 1.14, and 1.25 Ω, respectively, whereas the R_ct_ values were 0.23, 0.18, 0.16, 0.26, and 0.31 Ω, respectively. The BCS‐200 electrode exhibited the lowest R_s_ and R_ct_ values, indicating higher conductivity and fewer diffusion pathways at the electrolyte/electrode interface, which improves the electrochemical characteristics, as shown in Figure 3d. The CV profile of the BCS‐200 electrode is shown in Figure 3e. CV curves were obtained at different scan rates from 3 to 20 mV s^−1^ within a potential window of 0.0–0.6 V. When the scan rate increases, the peak current response also increases. Moreover, owing to the polarization effect, there is a shift in the oxidation and reduction peaks. Furthermore, the morphology of the BCS‐200 electrode provides a large contact area between the electrode and electrolyte, while the redox reactions of Cu^0^/Cu^2+^, Bi^+^/Bi^3+^, and Se^2−^ promote OH^−^ transfer in the electrolytes. As a result, the electrode exhibits significant capacitance improvement during the Faradaic process. A possible redox reaction was observed as follows.^[^ ^52^ ^]^

(7) $$Bi_{2}\mathrm{Cu}O_{12}Se_{4}+K^{+}+e^{-}↔K\left(Bi_{2}\mathrm{Cu}O_{12}Se_{4}\right)$$

The charge‐storage mechanism of the BCS‐200 electrode was analyzed using Equation (8).^[^ ^53^, ^54^ ^]^

(8) $$i_{p}=av^{b}$$

Here, *a* and *b* are constant parameters, *i_p_* is the peak current, and *v* is the scan rate. Based on the expected value of *b*, there are two possibilities: (i) for *b* = 1, the material displays a dominant surface capacitive contribution process, and (ii) for *b* = 0.5, the material shows a dominant diffusion contribution process. Herein, the BCS‐200 UTNS electrode exhibited a *b* value of 0.54, which is close to 0.5, thus indicating a diffusion‐controlled Faradaic nature, as shown in Figure 3f. From the CV curves, the capacitive/diffusion‐controlled contributions were calculated using Equation (9).^[^ ^55^, ^56^ ^]^

(9) $$i_{p}=k_{1}v+k_{2}v^{1/2}$$

where *k*_1_ and *k*_2_ are the variable paramters which are obtained from the plot of *v* ^1/2^ versus *i*/*v* ^1/2^. The capacitive and diffusion‐controlled current contributions of the BCS‐200 electrode are shown in Figure 3g. At 3, 5, 7, 9, 11, 15, and 20 mV s^−1^, the diffusion‐controlled contribution percentages were 96.1%, 95.1%, 94.2%, 93.5%, 92.9%, 91.8%, 90.6%, and 88.8%, respectively, indicating a dominant charge‐storage process. The GCD curves were measured at different current densities ranging from 2, 3, 5, 7, 10, 15, 20, 25, and 30 mA cm^−2^, as shown in Figure 3h. The GCD curves exhibited symmetry in the charge/discharge times, indicating good electrochemical reversibility. The presence of nonlinear discharge curves indicates that the resultant electrode material has battery‐type characteristics. In addition, the BCS‐200 electrode showed significant C_s_ values of 330.9 mAh g^−1^ at a low current density of 2 mA cm^−2^ and 163.2 mAh g^−1^ at a high current density of 30 mA cm^−2^, as shown in Figure 3i. Increasing the current density reduces the resultant C_s_ value because of a decrease in the associated ion‐exchange process. At a low scan rate, the OH^−^ ions from the aqueous electrolyte can take adequate time to diffuse into the electrode material. However, at high scan rates, the diffusion of ions makes it difficult for them to reach the inner layers of the entire material. Furthermore, the cycling stability of SCs is another vital criterion to be considered for real‐time applications. The cycling stability of the BCS‐200 electrode was investigated based on the GCD curves in 1 M KOH aqueous solution at 30 mA cm^−2^ as shown in Figure 3j. After the completion of 30000 GCD cycles, the BCS‐200 electrode maintained a capacity retention of 92.2% with 90.5% Coulombic efficiency. The Coulombic efficiency was calculated using Equation (S5) (Supporting Information). The ultra‐long stability is due to the long‐sustaining morphology and ultrahigh chemical and structural stability of the electrode. Most battery‐type materials currently exhibited a limited cycling life of <10000 cycles. The electrochemical performance of the BCS‐200 electrode, compared with previouse reports, is presented in Table S3 (Supporting Information). For the BCS‐200 electrode material, EIS analysis was performed before and after cycling, as shown in the inset of Figure 3j left side. Before and after the cycling test, the R_s_ values were 0.87 and 1.3 Ω, respectively. Also, the R_ct_ values were noted to be 0.16 and 4.1 Ω before and after the cycling test, respectively. In addition, the FE‐SEM images of the BCS‐200 UTNSs electrode before and after the cycling test are shown in the inset of Figure 3j right side. The UTNSs coarsened with the electrolyte solution during the continuous cycling process, as shown in the FE‐SEM images after the end of the stability test. In addition, the excellent electrochemical properties of the BCS‐200 UTNSs electrode material can be described based on its morphological characteristics, as shown in Figure 3k. The unique UTNSs morphology comprising small NSs provides several electroactive sites, shortens the ion diffusion distances, offers good electrical conductivity and mechanical strength, and maintains structural integrity during cycling. The electrochemical properties of the BCS‐100, BCS‐150, BCS‐250, and BCS‐300 were tested at different scan rates and current densities as shown in Figures S3 and S4 (Supporting Information).

**Figure 4:**
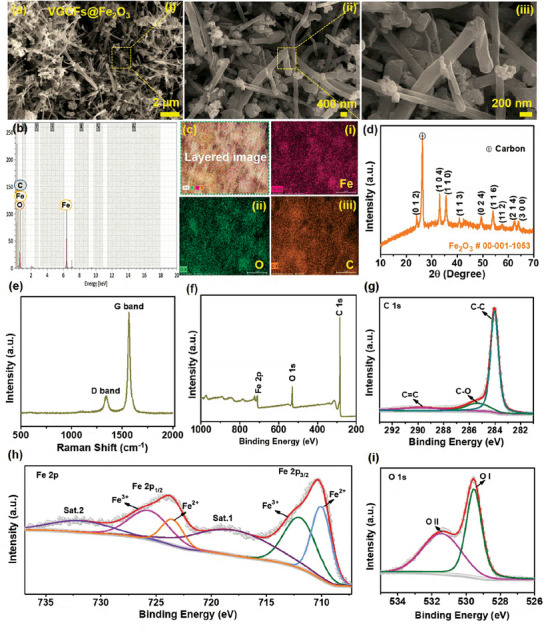
ai–iii) Low‐ and high‐magnification FE‐SEM images, b) EDX spectrum, and ci–iii) elements mapping images of the VGCFs@Fe_2_O_3_ composite material. d) XRD pattern and e) HR Raman spectrum of the VGCFs@Fe_2_O_3_ composite material. f) Total XPS survey scan spectrum and HR XPS core‐level spectra of the g) C 1s, h) Fe 2p, and i) O 1s elements.

The morphology of the prepared VGCFs@Fe_2_O_3_ composite negative electrode material was analyzed using the FE‐SEM images shown in **Figure** **4**a. The low‐magnification FE‐SEM image revealed an even distribution of VGCFs in the cluster of Fe_2_O_3_ spheres (Figure 4a(i)). The high‐magnification FE‐SEM images (Figure 4a(ii),(iii)) showed numerous VGCFs with incorporated Fe_2_O_3_ NSs. VGCFs may provide a large number of active sites and a high surface area, which is helpful for redox reactions and improves the electrochemical properties. In addition, the VGCFs serve as conductive bridges between Fe_2_O_3_ NSs. In addition, EDX spectroscopy analysis was carried out on the prepared VGCFs@Fe_2_O_3_ composite, and the resultant layered electronic image showed a uniform distribution of Fe, O, and C, as shown in Figure 4b. The colored images revealed the even dissemination of all the Fe, O, and C elements (Figure 4c(i)–(iii)). The FE‐SEM images of the Fe_2_O_3_ electrode at low and high magnifications are shown in Figure S5 (Supporting Information). The XRD analysis was used to study the phases obtained. The XRD peaks noted at the 2θ values of 24.1°, 33.1°, 35.7°, 40.8°, 49.4°, 54.0°, 57.7°, 62.6°, and 64.2° corresponding to the (012), (104), (110), (113), (024), (116), (112), (214), and (300) crystal planes, respectively are shown in Figure 4d. The diffraction planes are consistent with those of the Fe_2_O_3_ phase (JCPDS # 00‐001‐1053). Furthermore, a significantly intense peak can be seen at the 2θ value of 26.4° which represents the carbon in the prepared sample. These XRD results confirmed the formation of the VGCFs@Fe_2_O_3_ composite. The HR Raman spectrum of the VGCFs@Fe_2_O_3_ composite is shown in Figure 4e. In this spectrum, the “D” band at ≈1350 cm^−1^ and the “G” band at ≈1575 cm^−1^ correspond to the sp^3^ hybridization (carbon atoms of disordered graphite) and sp^2^ hybridization (carbon atoms of crystalline graphite), respectively.^[^ ^57^ ^]^ The elemental composition and valence state of the VGCFs@Fe_2_O_3_ composite were studied by HR‐XPS analysis. The total XPS survey spectrum revealed the presence of C, Fe, and O elements at the binding energies of 285.3, 711.2, and 530.2 eV, respectively as shown in Figure 4f. The atomic percentages of the VGCFs@Fe_2_O_3_ composite were estimated using XPS and EDX analyses (Table S2 (Supporting Information)). Both studies revealed nearly the same results. The C 1s, Fe 2p, and O 1s HR XPS profiles are shown in Figure 4g–i. The C 1s core‐level spectrum shown in Figure 4g consists of three subpeaks at 284.0, 285.4, and 289.9 eV which are related to the C–C, C–O, and C = O bonds, respectively.^[^ ^58^ ^]^ The Fe_2_O_3_ material was added to the VGCFs by ultrasonication process. The core‐level Fe 2p XPS spectrum is shown in Figure 4h. Two separate peaks were identified at ≈710.3 and 724.1 eV which correspond to the Fe 2p_3/2_ and Fe 2p_1/2_, respectively. The Fe 2p_3/2_ peak is again split into two peaks corresponding to Fe^3+^ and Fe^2+^ at ≈712.2 and 710.0 eV, respectively. Similarly, Fe 2p_1/2_ gave two peaks of Fe^3+^ and Fe^2+^ at ≈725.9 and 723.6 eV, respectively. In addition, two satellite peaks at ≈718.2 and 732.3 eV corresponding to Fe 2p_3/2_ and Fe 2p_1/2_, respectively are identified.^[^ ^59^, ^60^ ^]^ The core‐level O 1 s is shown in Figure 4i. The spectrum contains two main peaks at ≈529.5 and 531.3 eV which correspond to the lattice/chemisorbed O on the surface, respectively.^[^ ^37^, ^61^ ^]^

**Figure 5:**
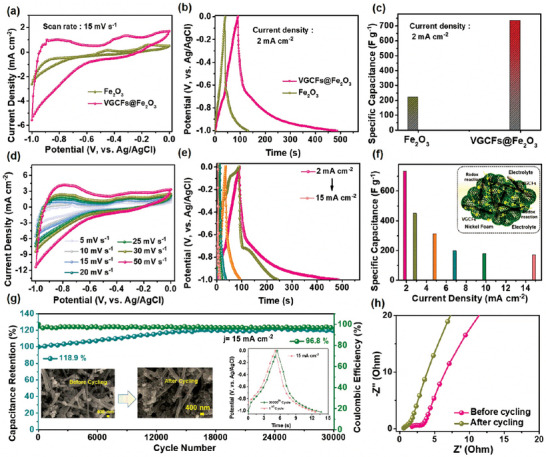
Comparative a) CV curves, b) GCD curves, c) C_sc_ values of the VGCFs@Fe_2_O_3_ composite and Fe_2_O_3_ electrodes, d) VGCFs@Fe_2_O_3_ composite CV curves from 5 to 50 mA cm^−2^, and e) GCD curves, and f) the C_sc_ values of the VGCFs@Fe_2_O_3_ composite. The inset of (f) shows the structural benefits of the VGCFs@Fe_2_O_3_ composite. g) Cycling stability and Coulombic efficiency results of the VGCFs@Fe_2_O_3_ composite electrode. The left inset of (g) shows the FE‐SEM images of the composite electrode before and after the cycling test. The right inset shows the initial and final cycle h) EIS plots before and after the cycling test for the VGCFs@Fe_2_O_3_ composite electrode.

The preparation process of the negative electrode slurry is discussed in Section SII (Supporting Information). The CV, GCD, and EIS properties of the VGCFs@Fe_2_O_3_ composite and Fe_2_O_3_ electrode materials are shown in **Figure** **5**. The CV curves were compared at a fixed scan rate of 15 mV s^−1^ as shown in Figure 5a. The prepared VGCFs@Fe_2_O_3_ composite electrode showed a dominant response in the CV region compared to the Fe_2_O_3_ electrode. Similarly, the GCD curves were compared at the current density of 2 mA cm^−2^ (Figure 5b). The VGCFs@Fe_2_O_3_ composite electrode exhibited longer charge/discharge times than the Fe_2_O_3_ electrode. VGCFs were added to Fe_2_O_3_ spheres to achieve these improvements, and the resultant morphology may provide a high surface area and several active sites at the electrode and electrolyte interface. Furthermore, for the VGCFs@Fe_2_O_3_ composite and Fe_2_O_3_ electrodes, the C_sc_ values were calculated at 2 mA cm^−2^, and the resultant values are plotted in Figure 5c. The C_sc_ value of the VGCFs@Fe_2_O_3_ composite electrode was 734.4 F g^−1^ and it was 221.7 F g^−1^ for the Fe_2_O_3_ electrode. In addition, the CV curves of the VGCFs@Fe_2_O_3_ composite electrode were tested at different scan rates ranging from 5 to 50 mV s^−1^, within the potential window of 0‐0.1 V as shown in Figure 5d. All the CV curves showed a combination of pseudocapacitive and electric double‐layer capacitive characteristics. Here, the electric double‐layer capacitive behavior was dominant over the pseudocapacitive behavior because of the high concentration of VGCFs in the Fe_2_O_3_ electrode material. The possible redox reactions of the VGCFs@Fe_2_O_3_ composite electrode involving Fe^3+^/Fe^2+^ are as follows^[^ ^62^ ^]^:

(10) $$Fe_{2}O_{3}+OH^{-}⇋Fe_{2}O_{3}\mathrm{OH}+e^{-}$$

In addition, the GCD curves were measured at the current densities of 2, 3, 5, 7, 10, and 15 mA cm^−2^, as shown in Figure 5e. All the GCD curves follow a trend similar to that of the CV curves. The C_sc_ values for the VGCFs@Fe_2_O_3_ composite electrode were noticed as 734.4, 449.1, 313.5, 199.7, 178.9, and 171.6 F g^−1^ at the current densities of 2, 3, 5, 7, 10, and 15 mA cm^−2^, respectively. At low current densities, the electrode material and electrolyte have sufficient time to react, resulting in high C_sc_ values. However, at higher current densities, redox reactions take place on the surface of the material, and thus the *C*_sc_ values decrease owing to limited ion diffusion. Moreover, the inset in Figure 5f shows the morphological advantages of the VGCFs@Fe_2_O_3_ composite electrode material, which includes spherical architectures with a large surface area that may provide several active sites for interaction between the electrolyte and electrode interface. The combination of spheres with conductive VGCFs supports fast ion transfer between the spheres and current collector. The VGCFs act as conductive bridges between the spheres. Moreover, the CV and GCD curves of the Fe_2_O_3_ electrode were obtained at different scan rates and current densities, as shown in Figure S6 (Supporting Information). Generally, cycling life is a vital factor for SCs. The cycling stability of the VGCFs@Fe_2_O_3_ composite electrode was tested at a current density of 15 mA cm^−2^. During this process, the K^+^ and OH^−^ ions start migrating and reacting with the internal sites of the nanostructures, resulting in increased retention after a long cycling process. After the completion of 30000 GCD cycles, the VGCFs@Fe_2_O_3_ electrode maintained a capacance retention of 118.9% with 96.8% Coulombic efficiency as shown in Figure 5g. The composite VGCFs@Fe_2_O_3_ electrode maintained higher capaciance retention and Coulombic efficiency, which indicates the structural stability of the prepared composite. It is observed that the capaciance of the material continues to increase with 118.9% capaciance retention, even after 30000 GCD cycles. This may be an advantage of the porous NSs and VGCFs mixed morphology, as can be seen in Figure 4. The NSs provide a large surface area, and the gradual rise in capacity retention can be attributed to the activation of the material due to the continuous diffusion of electrolyte within the open‐porous channels of the composite material, followed by complete wetting of the electrode material. The increase in the number of electroactive sites during the extended charge‐discharge cycles is primarily responsible for facilitating quick electrochemical redox reactions, which in turn enhances the cycling stability of the electrode.^[^ ^63^ ^]^ In addition, the integration of nanomaterials like carbon nanotubes with metal oxides into electrode structures increases conductivity and provides additional active sites for charge storage, thereby improving cycling stability.^[^ ^64^ ^]^ Herein, the VGCFs in composites are carbon‐based materials that are chemically stable, which is crucial for long‐term cycling stability.^[^ ^65^ ^]^ They can withstand repeated charge and discharge cycles without significant degradation, leading to a prolonged device lifetime. Therefore, the composite showed outstanding cycling stability with a remarkable capaciance retention of 118.9%. Similar behavior in cycling stability with excessive capacity/capacitance retention was also reported in previous literature.^[^ ^66^, ^68^ ^]^ Moreover, the initial and final cycles of the GCD curves were compared and plotted in the left inset of Figure 5g. From the GCD results, the charging and discharging times for the last cycle were higher when compared to the initial cycles, which further supports the superior capacitance retention of the composite. The FE‐SEM images of the VGCFs@Fe_2_O_3_ composite electrode material before and after the cycling test are shown in the right inset of Figure 5g. Furthermore, EIS analysis was performed for the electrode before and after the cycling stability test, and the resultant values are plotted in the Nyquist plot (Figure 5h). Before the cycling test, the R_s_ and R_ct_ values obtained are 1.43 and 1 Ω, respectively, whereas after the cycling stability test, the R_s_ and R_ct_ values are 2.9 and 0.75 Ω, respectively. A low R_ct_ value indicates that the electrode material has higher capacitive performance and higher ion diffusion. These results indicate that the achieved VGCFs@Fe_2_O_3_ composite material is a suitable negative electrode for SCs applications. The comparison of C_sc_/C_s_ values of the VGCFs@Fe_2_O_3_ negative electrode material with previously published reports is shown in Table S4 (Supporting Information).

**Figure 6:**
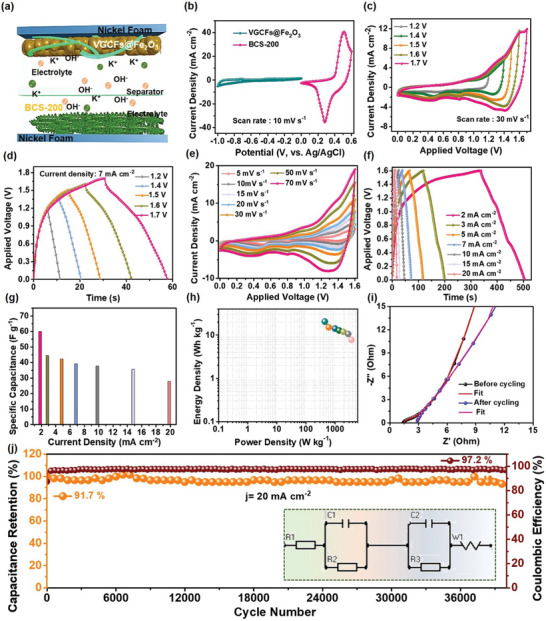
a) Schematic diagram of the SSASC cell. b) CV curves with reference to the potential windows for the BCS‐200 UTNSs and the VGCFs@Fe_2_O_3_ composite electrodes. c,d) CV and GCD curves at different voltage windows, e,f) CV at different scan rates, and GCD curves at different current densities for the SSASC cell. g) C_sc_ values at different current densities, h) Ragone plot, i) EIS plots before and after the cycling test for the SSASC cell. j) Cycling stability and Coulombic efficiency results. The inset of (i) shows the corresponding circuit diagram.

The practicality of the prepared electrode materials was tested by fabricating a semi‐solid‐state asymmetric SC (SSASC), as shown in **Figure** **6**. The SSASC cell was fabricated using the optimized BCS‐200 material as the positive electrode and the VGCFs@Fe_2_O_3_ composite as the negative electrode. The prepared PVA/KOH gel electrolyte was applied to the active area of both electrodes for 10 min. The two electrodes were then placed facing each other with a Whatman filter paper as a separator between them to avoid short circuits. It was then carefully packed using Parafilm, as shown schematically in Figure 6a. During the charge/discharge process, the negatively charged hydroxyl ions (OH^−^) move to the positive electrode side. Similarly, the positively charged potassium ions (K^+^) move toward the negative electrode. On the BCS‐200 electrode, oxidation reactions occur on the surface owing to charge separation at the positive electrode. Moreover, the 2D UTNSs of the BCS‐200 electrode provide a high surface area, which improves ion accommodation on the surface of the electrode. During the discharging process, the oxidation‐dissociated ions return to the electrolyte. Before the fabrication of the SSASC, the mass of both electrodes was calculated using Equation (S6) (Supporting Information). Their masses are 1.4 and 2.6 mg, respectively. The CV curves of both the VGCFs@Fe_2_O_3_ and BCS‐200 electrodes at a fixed scan rate of 10 mV s^−1^ are shown under the applied voltage windows of −1.0–0 and 0–0.6 V, respectively, as shown in Figure 6b. The CV curves were obtained at a constant scan rate of 30 mV s^−1^ by varying the applied voltage window from 0 to 1.2 to 0–1.7 V. There is no deviation in the curves at the 0–1.6 V voltage window, but at the applied voltage window of 0–1.7 V, the CV curves show a small deviation (Figure 6c). Similarly, at a constant current density of 7 mA cm^−2^, the GCD curves were measured for different applied voltage windows from 0–1.2 to 0–1.7 V as shown in Figure 6d. Here, a small deviation was observed at a voltage window of 0–1.7 V. Based on the CV and GCD curves, it is confirmed that the optimal applied voltage window of the SSASC cell is 0–1.6 V. Furthermore, the CV curves of the SSASC cell tested at different scan rates of 5, 10, 15, 20, 30, 50, and 70 mV s^−1^ revealed both quasi‐rectangular curves and battery‐type behaviors as shown in Figure 6e. Furthermore, the GCD curves were tested at an applied voltage window of 0–1.6 V at different current densities of 2, 3, 5, 7, 10, 15, and 20 mA cm^−2^ as shown in Figure 6f. At 2 mA cm^−2^, the SSASC cell shows nearly symmetrical GCD curves, indicating appropriate mass balancing of the electrodes. Moreover, the C_sc_ values were calculated from the same GCD curves to get the values of 60.1, 44.4, 42.3, 39.2, 37.8, 35.7, and 27.8 F g^−1^, respectively, as presented in Figure 6g. The Ragone plot for the SSASC cell is shown in Figure 6h, which reveals a maximum E_d_ of ≈20.5 Wh kg^−1^, with a maximum P_d_ of ≈3630.7 W kg^−1^. These were calculated using Equations (S3) and (S4) (Supporting Information). In addition, R_s_ and R_ct_ values before the cycling test are noted as 1.42 and 1.45 Ω, while after the cycling test, the R_s_ and R_ct_ values are seen to be 2.88 and 1.15 Ω, respectively as shown in Figure 6i. The cycling stability of the assembled SSASC is also a key feature for evaluating commercialization standard. The SSASC cell was tested at 20 mA cm^−2^ for 40000 GCD cycles, as shown in Figure 6j. Furthermore, the assembled SSASC cell exhibited ultra‐cycling stability with 91.7% capacitance retention and 97.2% Coulombic efficiency even after 40000 cycles. This excellent cycling stability may be due to the crystalline and porous morphologies of the materials. Moreover, the resulting EIS curves before and after the cycling stability test were fitted using the Ivium software, and the equivalent circuit is shown in the inset of Figure 6j. The circuit consists of R_s_, R1, R2, C1, C2, and W0 components. The first parallel connection, R1 and C1, represents the Faradaic charge transfer in the surface layer of the active materials, and the other parallel connections, R2 and C2, represent the Faradaic charge transfer resistance through the electrode/electrolyte interface and capacitance, respectively.^[^ ^69^ ^]^

**Figure 7:**
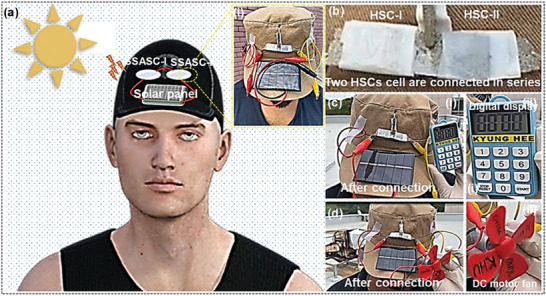
a) Schematic and photographic images of the real‐time feasibility test of the SSASC cells which were integrated on a cotton cap. b) Photographic image of the two SSASC cells connected in series connection. c,d) Photographic image for energizing different electronic components via the SSASC cell stored by the solar energy.

Practical applications of the fabricated SSASC cell were tested by powering different portable and wearable electronic components using green energy. So far, different forms of green energy, such as wind, solar, wave, and hydropower, have attracted attention for reducing the dependence on traditional fossil fuels. Among them, solar energy is clean and harmless and mostly uses renewable energy sources. In daily life, people farm in fields, walk to the office, play on the ground, and work on site. During these activities, solar energy can be effectively harvested and stored in SSASCs. The positive and negative terminals are connected to a commercial solar cell panel, as shown in the insets of **Figure** **7**a(i), to charge the SSASC cell. Photographs of two SSASC cells connected in series are shown in Figure 7b. The functionality of the SSASC cell was tested by connecting it to a digital display, as shown in Figure 7c(i),(ii). Furthermore, a small toy direct current motor fan is shown in Figure 7d(i),(ii). Overall, this ingenious, low‐cost, and environmentally friendly ED process can be used to fabricate innovative, highly sustainable, and high‐performance wearable energy storage devices.

## 3 Conclusion

In summary, a BSC‐200 electrode material with UTNSs morphology was prepared using a facile and low‐cost ED method, followed by calcination. The BCS‐200 electrode exhibited a C_sc_ value of 330.9 mAh g^−1^ at 2 mA cm^−2^, which is considerably higher than that of the other synthesized electrodes (BCS‐100, BCS‐150, BCS‐250 and BCS‐300). In addition, the cycling stability of the BCS‐200 electrode tested at a high current density of 30 mA cm^−2^ illustrates a high capacity retention of 92.2% after 30000 GCD cycles. Furthermore, a brief study was conducted on the deposition time by synthesizing four additional electrodes and comparing their performances with that of the optimized electrode. Besides, for the negative electrode, the VGCFs@Fe_2_O_3_ composite material was prepared using a solvothermal method. The composite electrode exhibited a high C_sc_ value of 734.4 F g^−1^ (C_s_:183.5 mAh g^−1^) at 2 mA cm^−2^. This was accompanied by an ultrahigh capacitance retention of 118.9%, even after 30000 cycles. Furthermore, the SSASC cell fabricated from these two electrodes attained a device voltage of 1.6 V combined with a C_sc_ value of 60.1 F g^−1^ at 2 mA cm^−2^. Moreover, the cell exhibited a maximum E_d_ value of 20.5 Wh kg^−1^ and a maximum P_d_ value of 3630.7 W kg^−1^, which is associated with a respectable cycling stability of 91.7% after 40000 cycles. Finally, the as‐fabricated SSASC cell was used to harvest solar energy effectively and thus power electronic devices. Consequently, synergetic interactions between the positive and negative electrodes may enhance the cycling stability and overall electrochemical performance of the SSASC cell. The proposed BSC‐200 UTNSs and VGCFs@Fe_2_O_3_ composite electrode materials are promising candidates for high‐performance, long‐lasting SC applications.

## 4 Experimental Section

All the chemicals and materials used in this study are described in the Supporting Information.

### Preparation of BCS‐200 Positive Electrode Material

The BCS‐200 material with UTNSs morphology was prepared using the chronoamperometric electrodeposition (ED) process on an NF substrate. Initially, the NF substrate (1 × 2 cm) was dipped in 1 M hydrochloric acid (HCl) and treated with an ultrasonic for 30 min. Later, the NF substrate was thoroughly washed with deionized (DI) water and ethanol to remove surface oxides and impurities and then dried in an oven for 12 h. On the other hand, the growth solution was prepared by adding 20 mM bismuth (III) nitrate pentahydrate (Bi(NO_3_)_3_·5H_2_O), 20 mM copper (II) nitrate hexahydrate (Cu(NO_3_)_2_·6H_2_O), 20 mM sodium selenite pentahydrate (Na_2_SeO_3_·5H_2_O), and 20 mM lithium chloride (LiCl) to 40 mL of the DI water until complete dissolution. After that, the solution was acidified by adding HCl until pH ≈3.5. The LiCl and HCl both act as structure‐directing salt and reducing agents, respectively. At room temperature, the ED process was carried out at a fixed voltage of −0.5 V for 200 s. For this process, the NF substrate was used as the working electrode, Pt wire was used as the counter electrode, and Ag/AgCl was used as the reference electrode. Later, the prepared BCS‐200 electrode material was washed with DI water and subsequently with ethanol, and it was dried in a vacuum oven for 10 min at 60 °C. The as‐obtained BCS‐200 electrode material was calcined in a muffle furnace in ambient air at 350 °C for 90 min with a ramp of 5 °C min^−1^. Finally, the BCS‐200 electrode material with UTNSs morphology was obtained. The mass of the BCS‐200 UTNSs material on the NF was noticed ≈1.41 mg cm^−2^. For comparison purpose, the growth time effect on the BCS morphologies was studied at different times such as 100, 150, 250, and 300 s using the above‐mentioned method, and the resulting electrode materials were named BCS‐100, BCS‐150, BCS‐250, and BCS‐300, respectively. The masses of the BCS‐100, BCS‐150, BCS‐250, and BCS‐300, electrodes were noticed as 1.33, 1.37, 1.63, and 1.8 mg cm^−2^, respectively.

### Preparation of Fe_2_O_3_ Electrode Material

The VGCFs@Fe_2_O_3_ composite material was prepared using a solvothermal process combined with a sonochemical method. Initially, 0.18 g of iron(III) nitrate nonahydrate (Fe(NO_3_)_3_·9H_2_O) and 0.12 g of HMTA (C_6_H_12_N_4_) were added to 50 mL of a mixed solution containing 5 mL of glycerol (C_3_H_8_O_3_) and 45 mL of isopropanol (IPA, CH_3_CHOHCH_3_) and the solution was stirred for 30 min. Next, this solution was transferred to an autoclave and dried at 180 °C for 12 h. The resulting powder was centrifuged with DI water and ethanol and was then dried in an oven at 90 °C for 12 h. Later, the optioned powder was calcined in a muffle furnace at 400 °C for 2 h and was referred to as Fe_2_O_3_.

### Preparation of VGCFs@Fe_2_O_3_ Negative Electrode Material

The VGCFs@Fe_2_O_3_ composite was prepared as follows. Initially, 150 mg of VGCFs were added to 20 mL of a DI water containing 50 mg of the prepared Fe_2_O_3_ powder. This mixture was ultrasonically treated for 1 h until it was completely dispersed to prevent the formation of lumps. The resulting powder was washed successively with DI water and ethanol by centrifuge, and was then dried. Finally, a VGCFs@Fe_2_O_3_ composite was obtained.

### Preparation of PVA‐KOH Gel‐Polymer Electrolyte

A PVA‐KOH gel electrolyte was prepared for the fabricated SSASC cell. Initially, 2 g of polyvinyl alcohol (PVA) was added to 20 mL of DI water, and the solution was stirred for 5 min. The PVA solution was then heated at 80 °C in an oil bath until completely dissolved in DI water. Next, a solution of 10 mL DI water containing 2 M KOH was added to the PVA solution, which was heated again to 80 °C while stirring. After cooling, the gel electrolyte was applied to both positive and negative electrodes.

## Conflict of Interest

The authors declare no conflict of interest.

## Supporting information

Supporting Information

## Data Availability

The data that support the findings of this study are available from the corresponding author upon reasonable request.
