# Supplementary material for: Rational Construction of Bi2CuO12Se4 and VGCFs@Fe2O3 Composite Electrodes for High‐Performance Semi‐Solid‐State Asymmetric Supercapacitors
Source: Small Methods. 2024 Jun 16;8(12):2400149. doi: 10.1002/smtd.202400149 (PMC11672177; doi:10.1002/smtd.202400149)
Supplement: Supplementary file 1 — Supporting Information [file SMTD-8-2400149-s001.docx]

**Supporting Information**

**Rational Construction of Bi_2_CuO_12_Se_4_ and VGCFs@Fe_2_O_3_ Composite Electrodes for High-Performance Semi-Solid-State Asymmetric Supercapacitors**

*Manchi Nagaraju, Bhimanaboina Ramulu, Shaik Junied Arba*z, *Edugulla Girija Shankar,* *Ampasala Surya Kiran, and Jae Su Yu**

*Department of Electronics and Information Convergence Engineering, Institute for Wearable Convergence Electronics, Kyung Hee University, 1732 Deogyeong-daero, Giheung-gu, Yongin-si, Gyeonggi-do 17104, Republic of Korea*

*Corresponding author. Email address: jsyu@khu.ac.kr (J. S. Yu)

**Section I**

- 1. **Chemicals and materials**

All the utilized chemicals and reagents used in our research were laboratory-grade without any additional purification. The deionized (DI) water of 18.0 MΩ cm resistivity was obtained from a Milli-Q water purification system. Bismuth (III) nitrate pentahydrate (Bi (NO_3_)_3_·5H_2_O), copper (II) nitrate hexahydrate (Cu(NO_3_)_2_·6H_2_O), sodium selenite pentahydrate (Na_2_SeO_3_·5H_2_O), lithium chloride (LiCl), and hydrochloric acid (HCl) were purchased from Sigma-Aldrich Co., South Korea. Iron (III) nitrate nonahydrate (Fe(NO_3_)_3_·9H_2_O), glycerol (C_3_H_8_O_3_), isopropanol (IPA) CH_3_CHOHCH_3_, and hexamethylenetetramine (C_6_H_12_N_4_) were purchased from Sigma-Aldrich Co., South Korea. The vapor-grown carbon fibers (VGCFs) were purchased from Sigma-Aldrich Co., South Korea. Moreover, the nickel foam (NF) was obtained from MTI Korea, South Korea. Super-P carbon black (C65, TIMCAL), N-methyl-2-pyrrolidone (NMP, C_5_H_9_NO), polyvinylidene fluoride (PVDF, -(C_2_H_2_F_2_)_n_-), potassium hydroxide (KOH), ethanol (C_2_H_5_OH), and ethylene glycol (C₂H₆O₂) were received from Dae-Jung Chemicals Ltd., South Korea, and they are used throughout the experiments.

**1.2. Material characterization techniques**

The surface morphology of all the prepared samples was studied by using a field-emission scanning electron microscope (FE-SEM, Carl Zeiss, LEO SUPRA 55, 5 kV). The crystallinity and phase purity of the prepared samples were investigated by X-ray diffraction (XRD, Cu Kα radiation, M18XHF-SRA, Mac Science) analysis at the 2θ range of 20-70° scanned for 4^o^ per minute with Cu kα (λ = 0.15406 nm) radiation. The energy-dispersive X-ray (EDX) spectroscopy was used to analyze the elements in the sample. To study the chemical composition and oxidation states of the elements in samples, X-ray photoelectron spectroscopy (XPS) (Al Kα radiation) and high-resolution Raman spectroscopy (HR Raman, WITec) were used to detect the presence of carbon and the corresponsing vibrations.

**Section II**

**1 Electrochemical measurements**

The electrochemical properties of cyclic voltammetry (CV), galvanostatic charge-discharge (GCD), and electrochemical impedance spectroscopy (EIS) for all the synthesized electrodes (BCS-100, BCS-200, and BCS-300) were tested at room temperature using a three-electrode setup. The reference electrode was Ag/AgCl, the counter electrode was Pt, the as-prepared samples were used as the working electrodes, and 1 M potassium hydroxide (KOH) was used as the aqueous electrolyte. In addition, the VGCFs@Fe_2_O_3_ composite and Fe_2_O_3_ materials were prepared as negative electrodes using the traditional slurry coating processes described below. First, a homogeneous slurry was prepared with a weight ratio of 80: 10: 10 for the active material, polyvinylidene difluoride, and conducting carbon (super-P carbon black), respectively. After all the materials were well ground using a pestle and mortar, the resulting slurry was coated on an NF active area of 1 cm × 1 cm and dried in a laboratory-grade oven at 90 °C for 6 h. Later, the dried NF was compressed at 10 MPa. The mass loadings of the VGCFs@Fe_2_O_3_ composite and Fe_2_O_3_ were noted as 1.4 and 1.23 mg cm^-2^, respectively. Electrochemical properties were measured at room temperature by using an IviumStat electrochemical workstation.

The specific capacity (C_s_), specific capacitance (C_sc_), power density (P_d_), and energy density (E_d_) were calculated using the following formulae^S1, S2^:

C_s_ = $\frac{I\times\Delta t}{m\times3.6}$ (S1)

where *I* is the applied current, *Δt* is the discharging time (s), and *m* is referred to as the mass of the active electrode (g).

C_sc_ = $\frac{I\times\Delta t}{m\times V}$ (S2)

E_d_ = $\frac{1}{2\times3.6}{Ccs (\Delta v)}^{2}$ (S3)

P_d_­ = $\frac{Ed\times3600}{\Delta t}$ (S4)

where *Δv* is the potential window, and *Δt* is the discharging time (s). The Coulombic efficiency (η) was calculated by the following equation:

η = $\frac{\mathrm{td}}{\mathrm{tc}}\times100$ (S5)

where ‘td’ and ‘tc’ are the discharge and charge times in second. The mass loadings of the positive electrode (BCS-200) and the negative electrode (VGCFs@Fe_2_O_3_/NF) were calculated using the following equation:

$m_{-}=\frac{Q_{+}\times m_{+}}{C_{-}\times\Delta V_{-}}$ (S6)

where *Q*_+_ and *m*_+_ are the charge (C) and mass (g) of the positive electrode, respectively; and ΔV_–_, C_–_, and m_–_ are the potential window (V), specific capacitance (F g^-1^), and mass (g) of the negative electrode, respectively.

The atomic percentages of the prepared BCS-200 material were found to be Bi (10.88%), Cu (25.38%), Se (31.09%), and O (32.65%) as shown in Table S1. The XPS survey scan spectrum confirmed the presence of the Bi 4f, Cu 2p, Se 3d, and O 1s elements with atomic percentages of 4.37%, 9.25%, 14.63 and 71.75%, respectively. Similarly, the atomic percentages of the prepared VGCFs@Fe_2_O_3_ composite were found to be Fe (8.82%), O (16.40%), and C (74.78%) as shown in Table. S2. Similarly, the total XPS survey scan spectrum VGCFs@Fe_2_O_3_ composite confirmed the presence of the Fe 2p, O 1s, and C 1s elements with the atomic percentages of 4.42%, 12.19%, and 83.4%, respectively.

**Table S1.** Atomic (%) and mass (%) of the prepared BCS-200 material.


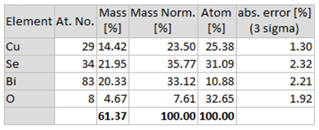


**Table S2.** Atomic (%) and mass (%) of the prepared VGCFs@Fe_2_O_3_ composite.


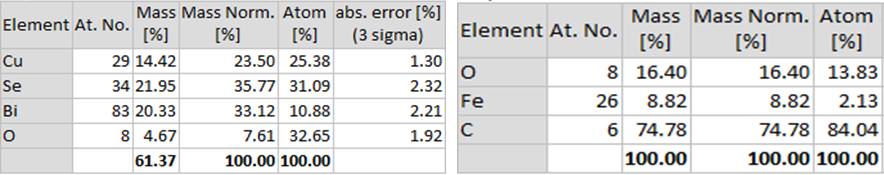


The surface morphologies of the BCS-100 and BCS-150 electrodes were examined by FE-SEM analysis (Fig. S1). Fig. S1(a)(i-iii) shows the low- and high-magnification FE-SEM images of the BCS-100, revealing uneven nanosheets (NSs) due to the shorter reaction time of 100 min, which results in underdeveloped NSs. The surface morphologies of the BCS-150 electrode are shown in Fig. S1(b)(i-iii), exhibiting overdeveloped NSs. When the deposition time was further extended to 150 s, the NSs became thicker owing to the accumulation of several NSs.


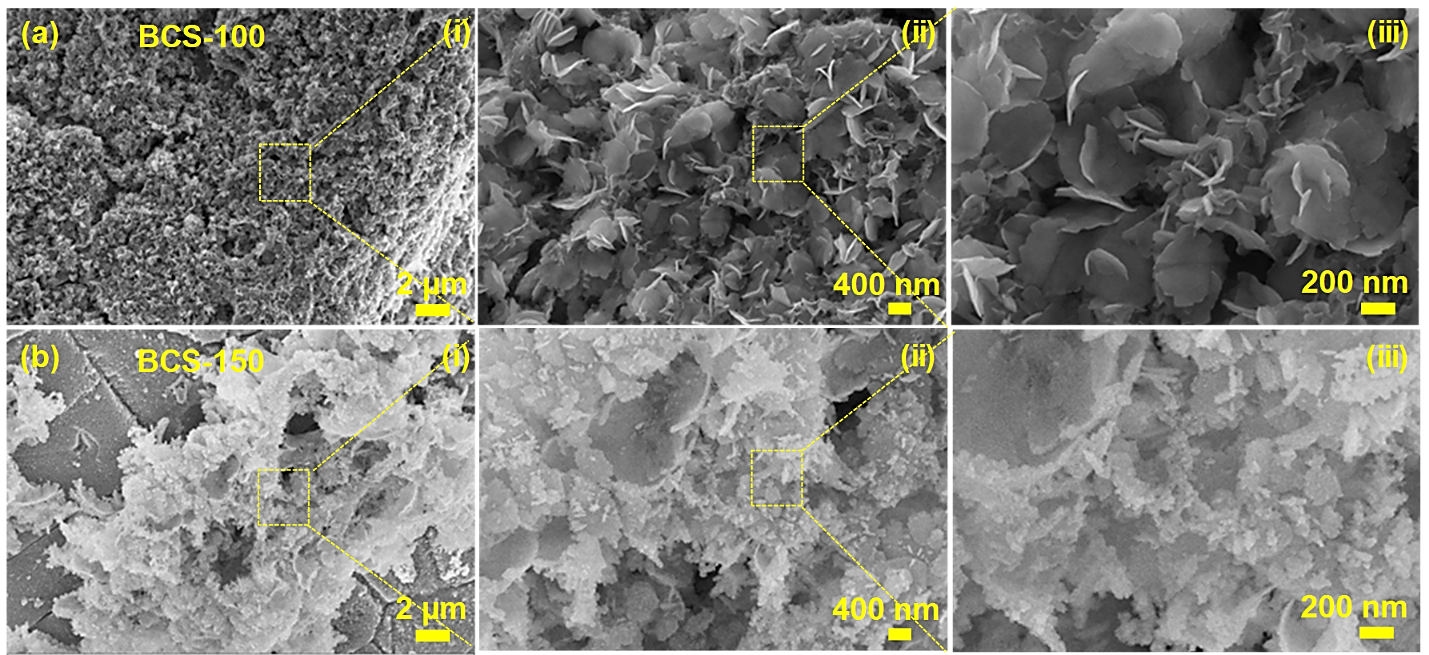


**Fig. S1.** (a) FE-SEM images of the BCS-100 material at (i-iii) low and high magnifications. (b) FE-SEM images of the BCS-150 material at (i-iii) low and high magnifications.

The surface morphologies of the BCS-250 and BCS-300 electrodes were examined using FE-SEM (Fig. S2). Fig. S2(c)(i-iii) shows the FE-SEM images of BCS-250, which reveals unform NSs. The surface morphologies of the BCS-300 electrode are shown in Fig. S2(d)(i-iii). From these images, the overdeveloped NSs were observed. The NSs became thicker owing to the accumulation of several NSs when the deposition time was increased to 300 s.


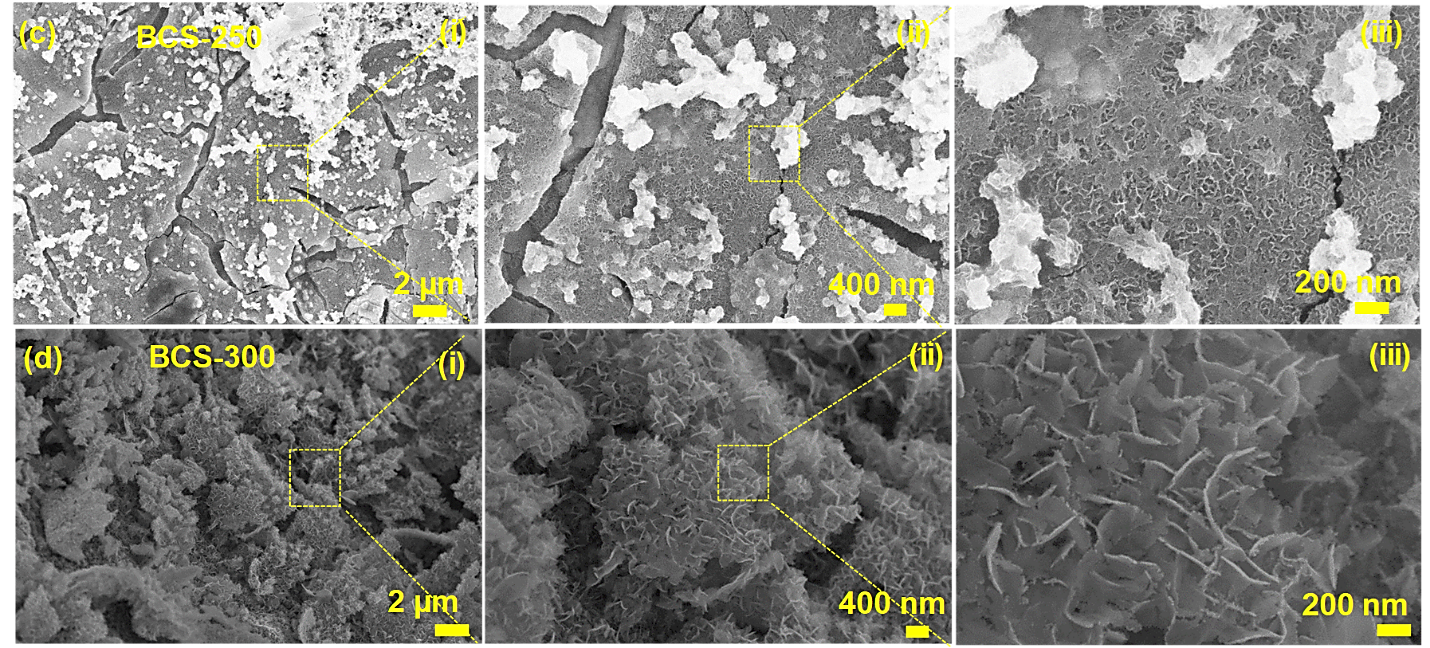


**Fig. S2.** (c) FE-SEM images of the BCS-250 material at (i-ii) low and (iii) high magnifications. (d) FE-SEM images of the BCS-300 material at (i-ii) low and (iii) high magnifications.

The CV curves of the BCS-100 electrode obtained at different scan rates are shown in Fig. S3(a). The current peaks shifted slightly as the scan rate increases from 3 to 20 mV s^-1^. In Fig. S3(b), the GCD curves of the BCS-100 electrode are displayed under the potential window of 0-0.55 V at different current densities of 2-30 mA cm^-2^. Based on the discharge time, the Cs values were calculated as shown in Fig. S3(c). The CV curves of the BCS-150 electrode obtained at different scan rates are shown in Fig. S3(d). Moreover, Fig. S3(e) displays the GCD curves of the BCS-150 electrode in the potential window of 0-0.55 V at different current densities at 2 to 30 mA cm^-2^. The C_s_ values at different current densities were calculated as shown in Fig. S3(f).


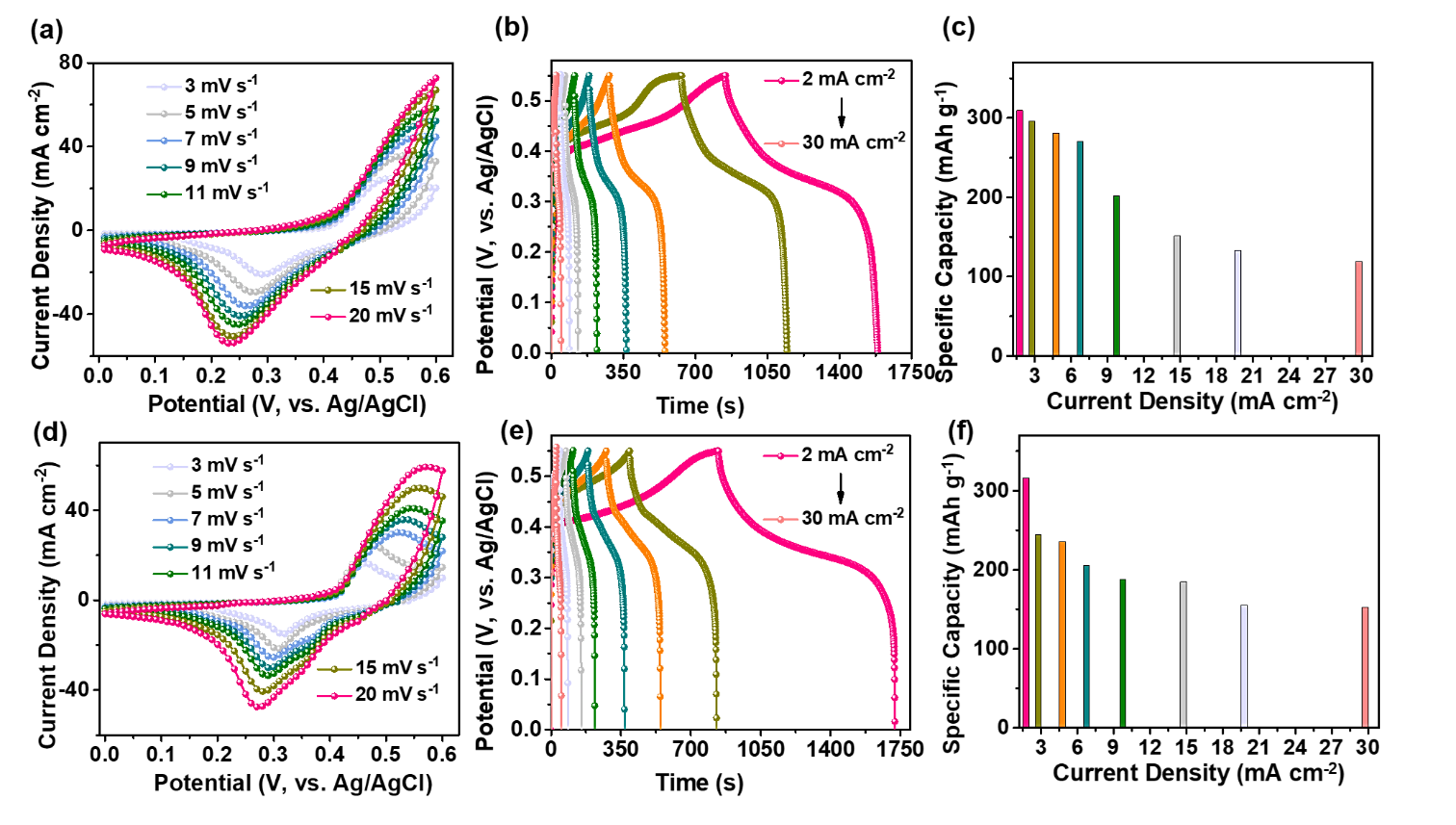


**Fig. S3.** (a) CV curves at various scan rates, (b) GCD curves at various current densities, and (c) C_s_ values at different current densities for the BCS-100 electrode. (d) CV curves at various scan rates, (e) GCD curves at various current densities, and (f) C_s_ values at different current densities for the BCS-300 electrode.

The CV curves of the BCS-250 electrode obtained at different scan rates are shown in Fig. S4(a). The current peaks shifted slightly as the scan rate increased from 3 to 20 mV s^-1^. Fig. S4(b) shows the GCD curves of the BCS-250 electrode at different current densities, ranging from 2 to 30 mA cm^-2^ under the potential window of 0-0.55 V. Based on the discharge time, the C_s_ values were calculated as shown in Fig. S4(c). The BCS-300 electrode CV curves at different scan rates from 3 to 20 mV s^-1^ are shown in Fig. S4(d). Moreover, the GCD curves of the BCS-300 electrode in the potential window of 0-0.55 V are shown at different current densities of 2-30 mA cm^-2^. The C_s_ values at different current densities were calculated and are shown in Fig. S4(f).


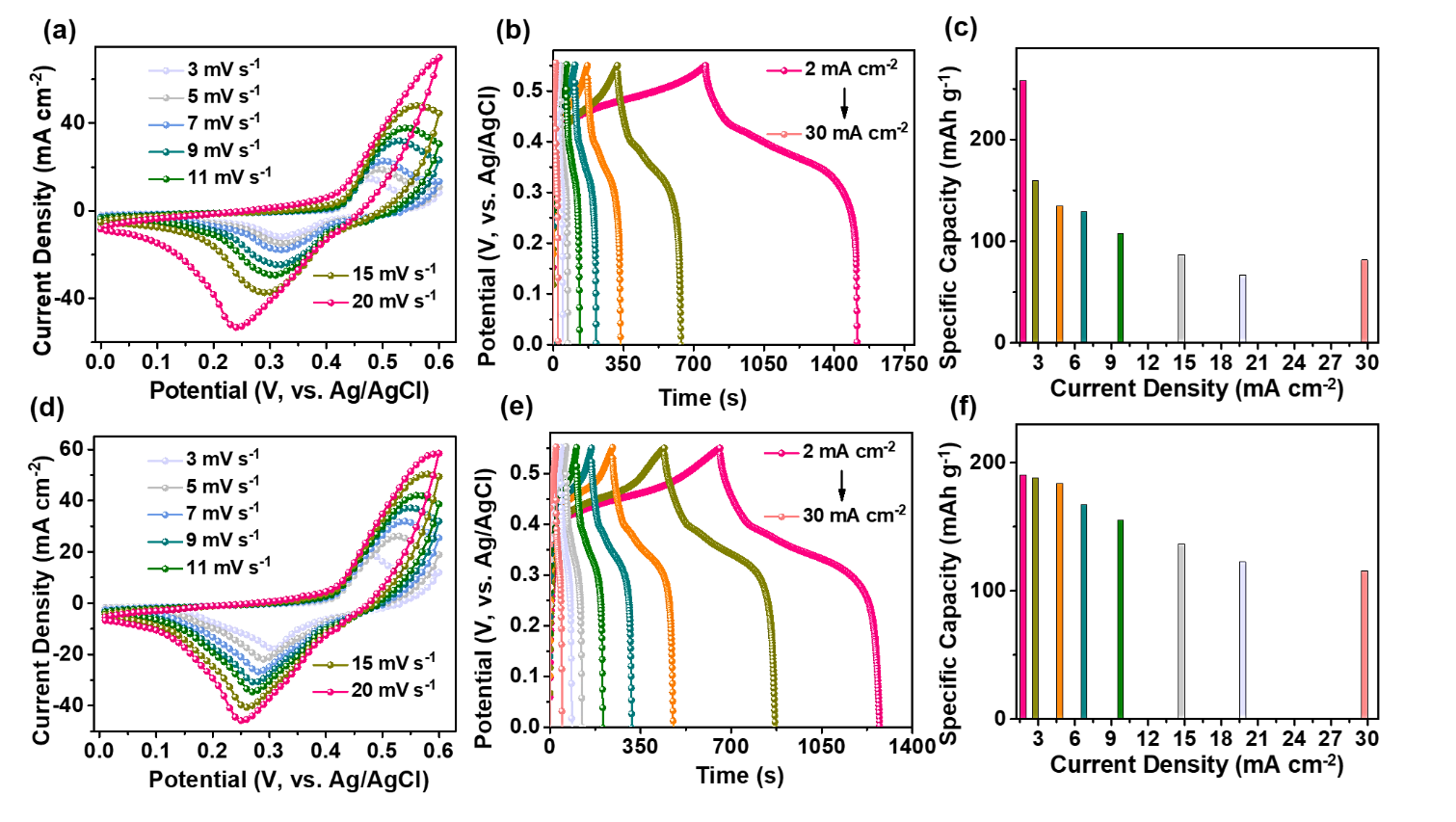


**Fig. S4.** (a) CV curves at various scan rates, (b) GCD curves at various current densities, and (c) C_s_ values at different current densities for the BCS-250 electrode. (d) CV curves at various scan rates, (e) GCD curves at various current densities, and (f) C_s_ values at different current densities for the BCS-300 electrode.

Fig. S5(a) and (b) shows the low- and high-magnification FE-SEM images of the prepared Fe_2_O_3_ material. In Fig. S5(c), the high-magnification FE-SEM image revealed that these spheres possess a rough surface area.


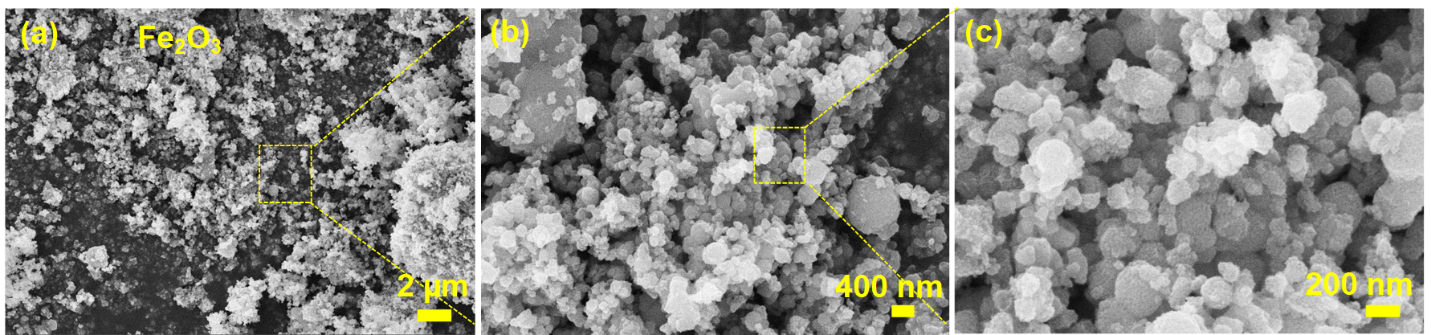


**Fig. S5.** FE-SEM images of the Fe_2_O_3_ material at (a,b) low and (c) high magnifications.

The CV curves of the Fe_2_O_3_ electrode at different scan rates from 5 to 50 mV s^−1^ are shown in Fig. S6(a). Furthermore, Fig. S6(b) shows the GCD curves of the Fe_2_O_3_ electrode within the potential window of -1 to 0 V at different current densities from 2 to 15 mA cm^-2^. Based on the discharge times, the C_sc_ values were calculated.


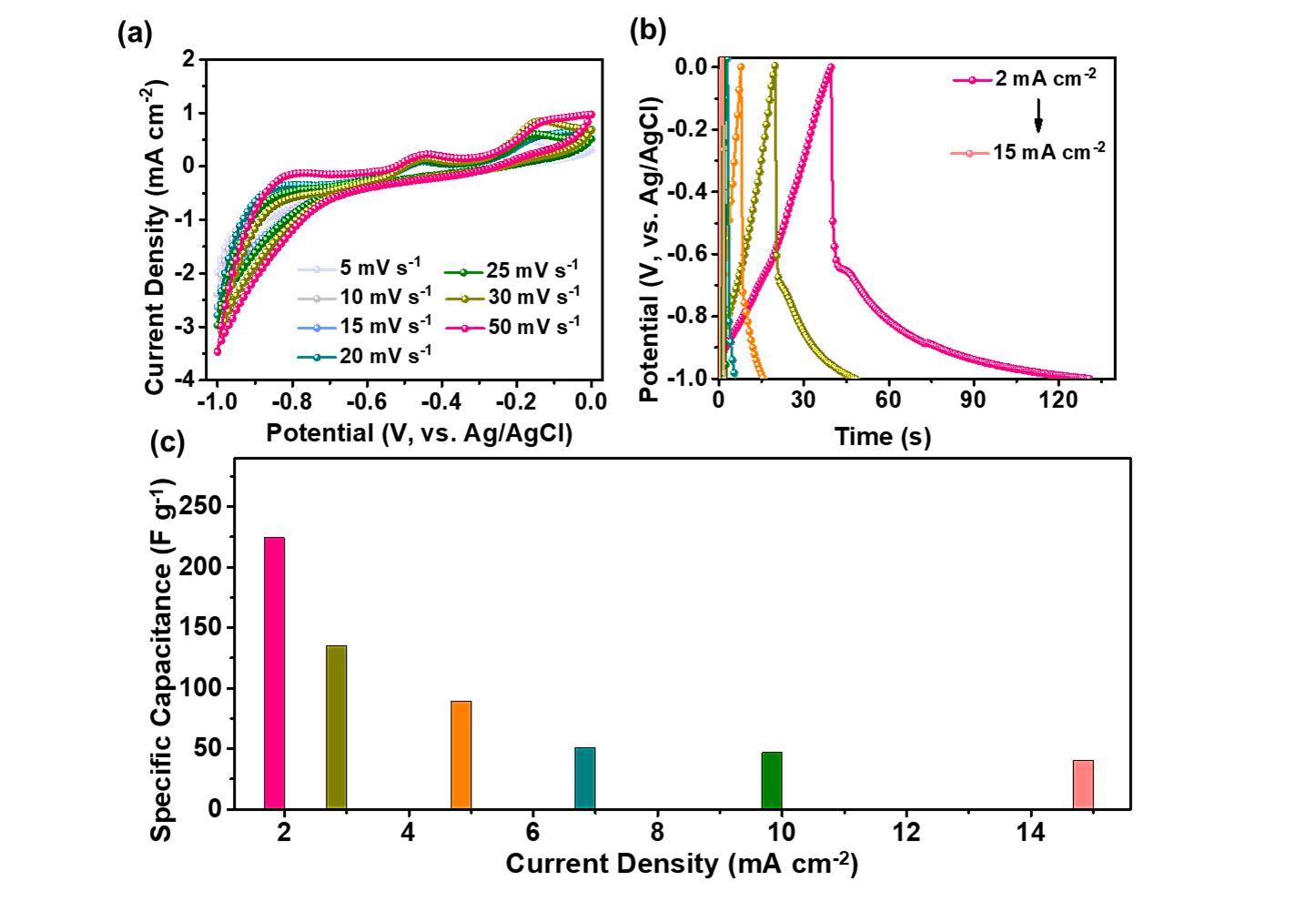


**Fig. S6.** (a) CV curves at various scan rates, (b), GCD curves at various current densities, and (c) C_sc_ values at different current densities for the Fe_2_O_3_ electrode.

**Table S3.** Comparative C_s_/C_sc_ values of the BCS-200 positive electrode material with previously published reports.

| Active material | Preparation method | Electrolyte | Specific capacity/ capacitance | Ref. |
| --- | --- | --- | --- | --- |
| Co_3_S_4_/CuS_2_/NiS_2_ | Solvothermal | 1 M KOH | 70.3 mAh g^-1^ | ^S3^ |
| N-GNTs@NiCoSe_2_/Ni_3_-Se_2_ | Electrodeposition | 2 M KOH | 1308 F g^-1^ | ^S4^ |
| NiCoSe_4_/N-rGO | Solvothermal | 2 M KOH | 120 mAh g^-1^ | ^S5^ |
| NiCoSe_4_/CFF | Microwave method | 6 M KOH | 1653.6 F g^-1^ | ^S6^ |
| MoS_2_/MoO_2_@CNT | Hydrothermal + Microwave | 6 M KOH | 228.4 F g^-1^ | ^S7^ |
| CuCo_2_Se_4_ | Hydrothermal | 3 M KOH | 512 F g^-1^ | ^S8^ |
| NiCu(OH)_2_@Ni-Cu-Se | Electrodeposition | 1 M KOH | 158.9 mAh g^-1^ | ^S9^ |
| BCS-200 | **Electrodeposition** | **1 M KOH** | **2206.6 F g^-1^/**  **330.9 mAh g^-1^** | **Our work** |

**Table S4.** Comparative C_sc_/C_s_ values of the VGCFs@Fe_2_O_3_ negative electrode material with previously published reports.

| Active material | Specific capacity | Electrolyte | Cycling stability/ retention | Ref. |
| --- | --- | --- | --- | --- |
| Ta-Cu_7_S_4_ | 675 F g^-1^ | 1 M KOH | 83.3% after 5000 cycles | ^S10^ |
| FeS_2_/Fe_2_O_3_@S-rGO | 790 F g^-1^ | 6 M KOH | - | ^S11^ |
| CuS/Fe_2_O_3_ | 921 F g^-1^ | 6 M KOH | - | ^S12^ |
| FeMn_2_O_4_ | 110 C g^-1^ | 2 M KOH | 84% after 1500 cycles | ^S13^ |
| MoS_2_/PPy | 350 F g^-1^ | 0.5 M Na_2_SO_4_ | 82% after 2000 cycles | ^S14^ |
| VN/NG | 445 F g^-1^ | 6 M KOH | 98.6% after 10000 | ^S15^ |
| Bi_2_O_3_/AC | 466 F g^-1^ | 6 M KOH | 70% after 3000 cycles | ^S16^ |
| VGCFs@Fe_2_O_3_ | **734.4 F g^-1^ (183.5 mAh g^-1^)** | **1 M KOH** | **118.9% after 30000 cycles** | **Our work** |

**References**

[S1] B. N. V. Krishna, O. R. Ankinapalli, A. R. Reddy and J. S. Yu, J. Mater. Sci. Technol. **2023**, 156, 230-240.

[S2] O. R. Ankinapalli, B. N. V. Krishna, Y. Hua and J. S. Yu, J. Alloys Compd. **2022**, 928, 167063.

[S3] A. R. Mule, B. Ramulu and J. S. Yu, Small, **2022**, 18, 2105185.

[S4] A. Meng, T. Shen, T. Huang, G. Song, Z. Li, S. Tan and J. Zhao, Sci. China Mater. **2020**, 63, 229-239.

[S5] Q. Yang, Q. Feng, X. Xu, Y. Liu, X. Yang, F. Yang, J. Li, H. Zhan, Q. Wang and S. Wu, Nanotechnology **2022**, 33, 345401.

[S6] L. Du, N. Lv, J. Li, J. Zhang, Y. Chen, Y. Zhang, Z. Li, X. Huang and J. Luo, J. Ind. Eng. Chem. **2024**, 120, 467-476.

[S7] Y. Tian, H. Du, M. Zhang, Y. Zheng, Q. Guo, H. Zhang, J. Luo and X. Zhang, J. Mater. Chem. C. **2019**, 7, 9545-9555.

[S8] F. Tavakoli, B. Rezaei, A. R. Taghipour Jahromi and A. A. Ensafi, ACS Appl. Mater. Interfaces **2020**, 12, 418-427.

[S9] V. T. Chebrolu, B. Balakrishnan, V. Raman, I. Cho, J.-S. Bak, K. Prabakar and H.-J. Kim, Appl. Surf. Sci. **2020**, 506, 145015.

[S10] X. Han, D. Zhang, Y. Qin, X. Kong, F. Zhang and X. Lei, Chem. Eng. J. **2021**, 403, 126471.

[S11] R. Bu, Y. Deng, Y. Wang, Y. Zhao, Q. Shi, Q. Zhang, Z. Xiao, Y. Li, W. Sun and L. Wang, ACS Appl. Energy Mater. **2021**, 4, 11004-11013.

[S12] X. Han, J. Ge, J. Luo, Y. Wang, X. Zhao, F. Zhang and X. Lei, J. Alloys Compd. **2022**, 916, 165443.

[S13] S. Nagamuthu, S. Vijayakumar, S.-H. Lee and K.-S. Ryu, Appl. Surf. Sci. **2016**, 390, 202-208.

[S14] Y. Chen, W. Ma, K. Cai, X. Yang and C. Huang, Electrochim. Acta **2017**, 246, 615-624.

[S15] J. Balamurugan, G. Karthikeyan, T. D. Thanh, N. H. Kim and J. H. Lee, J. Power Sources **2016**, 308, 149-157.

[S16] J. Li, Q. Wu and G. Zan, Eur. J. Inorg. Chem. **2015**, 2015, 5751-5756.
